# Strengths-Based Job Crafting and Employee Creativity: The Role of Job Self-Efficacy and Workplace Status

**DOI:** 10.3389/fpsyg.2021.748747

**Published:** 2021-12-15

**Authors:** Zheng Yang, Pingqing Liu, Zunkang Cui

**Affiliations:** School of Management and Economics, Beijing Institute of Technology, Beijing, China

**Keywords:** strengths-based job crafting, job self-efficacy, workplace status, employee creativity, self-affirmation theory

## Abstract

While argued to be fostering creativity, the effect of job crafting on creativity often turned out to be less effective than expected. The reason is that most existing studies focused on the top-down job design interventions. We proposed an elaborated theoretical model to explain the influence of strengths-based job crafting (SJC) on employee creativity (EC). Specifically, we examined the mediating effect of job self-efficacy (JSE) and the moderating effect of workplace status (WP) based on self-affirmation theory. A sample of 480 employees and their supervisors completed a battery of questionnaires. The results revealed that strengths-based job crafting was positively related to employee creativity, with job self-efficacy acting as a mediator for this relationship. Workplace status moderated both the direct and the indirect effects of job self-efficacy. For employees with a higher workplace status, strengths-based job crafting may generate more forces to promote employee creativity. The results suggest that strengths-based job crafting and workplace status can inspire employee creativity through a self-affirmation process.

## Introduction

Organizations increasingly rely on their employees to exhibit creativity at work. Employee creativity (EC), defined as the generation of novel and useful ideas (Shalley et al., 2004), is an important asset for organizations to deal with challenges and remain competitive. How to enhance employee creativity is a focal concern for academics and practitioners (Amabile et al., 2005; Rietzschel et al., 2016; Wu et al., 2020). The self-management practice of job crafting, or the changes that employees make to their jobs, has been proved to be an important antecedent of employee creativity (Bruning and Campion, 2018; Sun et al., 2020; Wang and Lau, 2021). However, most existing studies focused on the top-down job design interventions, which are generally found to be less effective than researchers and practitioners hope (Nielsen et al., 2010). The possible reason for this phenomenon may be that they follow the philosophy of “one size fits all” rather than the philosophy of “which size fits you?”

In the current study, we argue that strengths-based job crafting (SJC), a typical bottom-up job redesign, will stimulate employee creativity. Job crafting toward strengths refers to the self-initiated changes that individuals make in the task boundaries of their work to make better use of their strengths (Kooij et al., 2017). Ever since the concept of job crafting was first proposed by Demerouti et al. (2001), scholars have published a large number of studies on this subject, focusing on changing job demands and job resources (Wrzesniewski et al., 2013; Demerouti et al., 2015; Bruning and Campion, 2018). However, our current understanding only focuses on the “job” part, and ignores the “person” part. In other words, the existing research only captures on the superficial representation of job crafting (Zhang et al., 2021), and does not consider how to incorporate employees’ motives, strengths, and passions in the job crafting concept. In fact, the premise of job crafting is to alter the job characteristics according to their initiative. The self-initiated changes caused by personal strengths help employees to revise their work identities (Dierdorff and Jensen, 2018; Sun et al., 2020), and enhance the meaning of work through job crafting. Moreover, the freedom or discretion individuals have within the job constraints determines the perceived opportunities and internal motivation factors to generate new ideas (Wrzesniewski et al., 2013). Therefore, it is of great significance to consider the strengths-based job crafting in the formation of creativity.

Specifically, we focus on the relationship between strengths-based job crafting and employee creativity. Individual strengths are the personal characteristics that enable individuals to achieve their best performance (Wood et al., 2011). The goal of strengths-based job crafting is to change the parameters of one’s job for better use of individual strengths. According to Cohen and Sherman (2014), self-affirmation characterizes a self-system of psychological and behavioral tendencies in which how people see themselves motivates them to behave in ways to strengthen their self-efficacy beliefs. In other words, we examine the possibility of whether strengths-based job crafting can indirectly and positively impact employee creativity. Understanding the trigger mechanism of self-efficacy beliefs will be conducive to the theoretical and practical implications, including overall work efficiency and general management effectiveness improvement.

## Literature Review and Hypotheses

### Research on Strengths-Based Job Crafting

For more than 2decades, job crafting – defined as “the physical and cognitive changes individuals make in the task or relational boundaries of their work”(Wrzesniewski and Dutton, 2001) – has received increasing attention from scholars (Leana et al., 2009; Petrou et al., 2012; Ding et al., 2020; Knight et al., 2021). Our current understanding of the concept of job crafting was proposed by Wrzesniewski and Dutton (2001), Tims et al. (2012), and Bruning and Campion (2018) extended this theory. From a demand-resource perspective, job crafting was defined as “the changes that employees may make to balance their job demands and job resources with their abilities and needs” (Tims and Bakker, 2010). Job crafting represents actions that lead to changes in employees’ work beliefs, makes it an effective supplement to management (Lichtenthaler and Fischbach, 2019). As a concept reflecting employees’ proactive and voluntary adaptation to changes in the workplace, there are mainly two conceptualizations of role- and resource-based job crafting in the literature (Lichtenthaler and Fischbach, 2019). In the role-based job crafting conceptualization, employees tend to change the task and relational work role boundaries and their role perceptions at work (Wrzesniewski and Dutton, 2001). Employees develop their particular interpretation of their jobs, tasks, and social interactions with others. On the other hand, in the resource-based job crafting conceptualization, employees are inclined to pursue the increase of their work resources or the decrease of their challenging job demands. Scholars have formed several job crafting measurements based on the job crafting theory of Wrzesniewski and Dutton (2001). Among them, general and daily measurements of Petrou et al. (2012) were most frequently used. In addition, based on the JD–R model, Tims and Bakker (2010) classified job crafting into three dimensions: increasing job resources, increasing challenging goals, and decreasing hindering job demands. In recent studies, Bruning and Campion (2018) developed a role–resource approach–avoidance taxonomy that integrated and extended the dominant role- and resource-based perspectives of job crafting. In both job crafting types, employees seek to reshape their jobs to achieve better performance.

Strengths-based job crafting was conceptualized as the self-initiated changes within the work constraints to make better use of their strengths. Personal strength refers to the unique characteristics of an individual to perform best (Wood et al., 2011), and allow him or her to be energized, keep learning, and have peak experiences (Brewerton and Brook, 2010). Releasing individual strengths makes the employees authentic and productive, and crafting the job gives them a sense of meaning, identity, and calling. The underlying reasoning is that individual strengths become work-related characteristics to satisfy their need for personal control, positive identity, and emotional connections (Baumeister and Leary, 1995). Strengths-based job crafting is considered to be a kind of self-initiated change behavior designed to align the job with employees’ preferences, motivations, and passions. Crafting to better align one’s personal strengths makes it easier for individuals to be successful at work. According to Bouskila-Yam and Kluger (2011), strengths are considered as the ability to provide consistent, near-perfect performance. Although employees may craft their jobs in three different ways, the strengths-based job crafting makes it easier for the formation of new ideas. The bottom-up design of their work characteristics can increase the structural resources, social support, and challenging demands, and reduce the hindering demands (Wang and Lau, 2021). However, as creativity has the essential characteristic of agentic, the internal mechanism of job remodeling affecting creativity has not been fully revealed. Therefore, to better capture the role of strength in job crafting and how strengths-based job crafting influences employee creativity, we draw from the social cognitive theory and creativity literature, suggesting our theoretical model.

### Strengths-Based Job Crafting and Employee Creativity

As a typical extra-role performance above generally expected levels, creativity has received considerable attention in the field of organizational behavior research. The creative process model of Amabile (1996) defined creativity as the generation of new and useful ideas or products. The novelty of ideas or products is usually formed in the individual positive psychological processes, and the best ideas often come from unexpected sources (Astola et al., 2021). Prior research has indicated that employee creativity can be triggered by factors related to their self-awareness (Liu et al., 2016). Among them, strength use as a self-aware factor can influence employees’ creativity along with individual and contextual factors in the workplace (Kooij et al., 2017). Recent theorizations by support a more comprehensive view of strength use, by integrating energy, authenticity, and concentration as the factors related to employee performance (Dubreuil et al., 2014).

In current study, we argue that strengths-based job crafting, as one of the most important external manifestations of self-awareness, can stimulate employees’ creativity. First, crafting the job according to personal strengths can make employees energetic, productive, and satisfied (Dubreuil et al., 2014; Chon and Sitkin, 2021). That is, when employees engage in strengths-based job crafting at work, they will feel competent and vital. This heightened feeling of energy allows employees to generate more divergent thinking and work more vigorously and for longer periods, which is the important premise for the formation of individual creativity. Second, strengths-based job crafting enables employees to form a sense of control, autonomy, and authenticity in the workplace (Linley and Harrington, 2006). Authenticity allows employees to be themselves, and follow their work aspirations or directions. This kind of cognitive and psychological process is more conducive for employees to generate a feeling of being true to themselves (Ozer and Zhang, 2021), which determines the quantity and quality of creative ideas. Then, job crafting toward personal strengths encourages employees to experience a state of deep concentration at work, which is similar to flow (Schutte and Malouff, 2020). Concentration on tasks effectively stimulates the intrinsic motivation of employees and the pleasure of completing the work process. Therefore, strengths-based job crafting behaviors promote creativity through a range of positive work experiences. From such premises, we hypothesized that:

*H1*: Strengths-based job crafting is positively related to employee creativity.

### The Mediation Effect of Job Self-Efficacy

Strengths-based job crafting will affect employees’ job self-efficacy (JSE). Prior research has suggested that individuals with more strengths are more likely to generate beliefs in stimulating change (Tierney and Farmer, 2011). According to self-affirmation theory, they are more likely to take risks and seek opportunities for breakthroughs (Mao et al., 2021). This viewpoint is supported by groundbreaking research on self-efficacy of Bandura (1977). In the social cognitive theory, he proposed four methods of increasing efficacy expectations: performance accomplishments, emotional arousal, vicarious experience, and social persuasion. First, strengths-based job crafting can contribute to the generation of self-affirmation for individual performance accomplishment, which was considered to be the most effective method of building self-efficacy. Second, the positive emotional arousal caused by personal strength was found to increase self-efficacy, for the increased perceptions of self-competence and decreased perceptions of goal difficulty. Furthermore, the vicarious experience and social persuasion were found to help individuals create self-inducements to persist in their efforts, which was also associated with higher self-efficacy.

According to self-affirmation theory, individuals tend to behave in ways that strengthen their values and beliefs (Cohen and Sherman, 2014; Mao et al., 2021). As a belief originated from self-knowledge, self-efficacy refers to people’s sense of personal efficacy to exercise some control over events that affect their lives ((Bandura, 1997, 2012). People’s convictions in their own effectiveness are likely to affect whether they will even try to cope with given situations (Bandura, 1977). In other words, job self-efficacy reflects an individual’s confidence in whether he or she can use structural and cognitive resources to complete the task, which is an important psychological factor to produce new ideas. For example, individuals with low self-efficacy often treat challenges as a source of threat or limitations and doubt their ability to cope with. They will tend to adopt defensive withdrawal behavior to deal with the challenges. On the contrary, the ones with higher levels of job self-efficacy will set more challenging goals and be more likely to make persistent efforts to solve problems. They regard challenges as opportunities to realize self-worth, and adopting novel ideas to solve problems as an achievement. During this process, a strong sense of self-efficacy is necessary to the generative and exploratory processes for new ideas. Therefore, we expect job self-efficacy positively relates to employee creativity. In general, individuals who demonstrate more strengths-based job crafting, are more likely to be enthusiastic about creative exploration (Wisse et al., 2015). As such, they attend to have higher job self-efficacy, driving them to demonstrate more creativity. Therefore, we hypothesized that:

*H2*: Job self-efficacy mediates the relationship between strengths-based job crafting and employee creativity.

### The Moderating Role of Workplace Status

Workplace status (WP) is highly salient and of great importance in organizations (Chen et al., 2012; Djurdjevic et al., 2017). As a socially constructed subjective assessment, workplace status depends on coworkers’ collective beliefs, characterized by respect, admiration, and freely conferred deference (Anderson et al., 2015). According to the symbolic interactionist perspective, people make sense of whom they are based on their interactions with others (Djurdjevic et al., 2017). High workplace status reflects a positive organizational identity, indicating the recognition for one’s ability and the expectations for his or her performance (Lount et al., 2019). More accurately, although workplace status is conferred by others, it can strengthen how employees view their ability which further affects their internal belief systems. For example, individuals with a higher workplace status tend to be treated more fairly and receive more help to overcome obstacles, which is critical for them to produce novel and potentially useful results.

**Figure 1:**
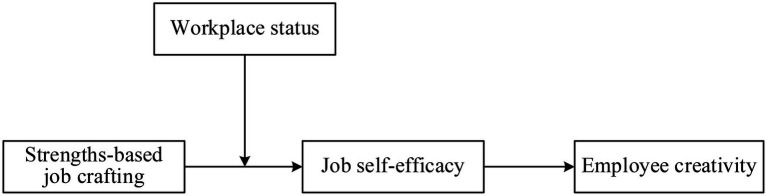
Theoretical model.

Therefore, we surmise that workplace status will affect the relationship between strengths-based job crafting and job self-efficacy, because the function of workplace status is associated with the nature of social enhancement processes. From a social enhancement perspective, the self-affirmation process caused by personal strengths depends on whether their socioemotional needs are fulfilled. High-status employees perceive their strengths more positively than low-status members because of the high social affirmation from the organization (Lee and Jeung, 2018). As mentioned above, workplace status is a subjective assessment conferred by others and can have an impact on individual beliefs (Djurdjevic et al., 2017; Lount et al., 2019). Individuals with a high workplace status are more likely to interpret the status signaling as an affirmation of their unique and outstanding strength, thus further enhancing their job efficacy beliefs stimulated by the strengths-based job crafting. This is particularly true for the star employees because they will seek to affirm their positive self-image and outstanding (Kehoe et al., 2018). On the contrary, employees with a low workplace status are likely to evolve into a denial of their ability to present novel ideas and weaken their self-beliefs, thus further restricting their creativity. Understanding when strengths-based job crafting is more likely to elicit job self-efficacy helps explicate the relationship between strengths-based job crafting and creativity. When strength leads to job self-efficacy and these elevated self-beliefs produce creativity, there will be a positive indirect effect between strengths-based job crafting and creativity through job self-efficacy. In summary, the impact of strengths-based job crafting on creativity through creative efficacy beliefs is likely affected by workplace status. Accordingly, we propose a first-stage moderated mediation model whereby the indirect effect between strengths-based job crafting and creativity through job self-efficacy is moderated by workplace status (see Figure 1).

*H3*: Workplace status will positively moderate the impact of strengths-based job crafting on job self-efficacy. Strengths-based job crafting has a stronger positive effect on job self-efficacy when workplace status is higher rather than lower.

*H4*: Workplace status will positively moderate the indirect relationship between Strengths-based job crafting and employee creativity via job self-efficacy, such that the indirect link will be stronger when workplace status is higher rather than lower.

## Materials and Methods

### Procedure and Participants

The data for this study were collected in 95 Chinese innovation-oriented technology enterprises companies. There were 105 participants and most of them were middle or junior managers with rich management experience. During the survey, these participants are taking an 8-week MBA course at a prestigious university in Beijing, China. These participants and their team members attach great importance to thinking and innovation at work. Before the survey, we informed the participants that the survey had nothing to do with their final grade in the course, and they could voluntarily choose to participate or not. At the start of the survey (week 1), we collected the basic information of the participants and their teams. A total number of 105 supervisors, holding jobs, such as manager, director, and administrator took part in our survey. All the participants joined the same WeChat group and received a link for the first online questionnaire sent by the researchers. We distributed random amounts of WeChat Lucky Moneys in the group to motivate the participants. Through this process, we obtained the participants’ demographic information, educational background information, work tenure, job characteristics, and so on.

Then, in order to make the data obtained in this study more authentic, we spent 2weeks establishing a good interactive relationship with them. In the second step (week 4), we invited them to select at least five of their subordinates to participate in our research. We designed and printed paper questionnaires, numbered them, and put them in envelopes. Participants took the envelopes back to the company for subordinates to fill out, sealed them, and handed them back to the researchers. In order to ensure the credibility of the research, we informed them that this survey is only for scientific research and there will be no disclosure of their personal information. In the third step (week 7), after we sorted out and entered the questionnaires of the subordinates of the participants, we designed and printed the questionnaires of the supervisors. The purpose of this is to invite supervisors to evaluate the performance and behavior of employees, improving the objectivity and accuracy of measurement. Subsequently, we manually matched the questionnaires filled in by supervisors with those filled by subordinates. In this survey, a total number of 105 questionnaires for supervisors and 480 questionnaires for subordinates were distributed. Finally, after kicking out the invalid questionnaire, 87 questionnaires for supervisors and 418 questionnaires for subordinates left, with effective recovery rates of 82.86 and 87.08%, respectively.

### Measures

In order to ensure the accuracy of the quantitative analysis, all the scales we used in this study had been published in authoritative journals, and generally had high reliability. As the original scale language is English, we used a method of translated and back-translated procedure to ensure accuracy of verbal expression in measurement. We invited two doctoral candidates in organizational behavior and one master’s student English Language Major to adjust the scales according to our research situation. After the overall design was completed, we invited two professors majoring in business administration to make comments and suggestions for our questionnaire. Then, we made the adjustment according to the expert’s suggestion. In addition, all items use a seven-point Likert scale (1 = strongly disagree and 7 = completely agree) in the survey.

#### Strengths-Based Job Crafting

As a kind of behavior aimed at adapting job to match the personal resources of the employee, it is more suitable to use the scale of personal subjective statement. In this study, we measured strengths-based job crafting using the four items developed by Kooij et al. (2017). Participants were asked how they interpreted the tasks that suit or not suit their strengths. A sample item is, “I organize my work in such a way that it matches my strengths” (*α* = 0.924).

#### Job Self-Efficacy

We measured job self-efficacy using the four items adapted from Jones (1986), and used the expressions of the items adjusted by Wilk and Moynihan (2005). The scale was designed to measure individuals’ self-confidence, or beliefs in their own competence, to meet the job demands in organizations. A sample item is, “I am confident that I am able to successfully perform my current job” (*α* = 0.895).

#### Employee Creativity

To avoid common method variance, we invited the supervisors to assess their subordinates’ creativity using a seven-item scale developed by Gong et al. (2009). Before sending out the questionnaires, we informed the supervisors of the purpose of our survey and the definition of creativity. A sample item is, “He or she often comes up with creative solutions to problems.” Based on the confirmatory factor analysis (CFA) results and interviewee characteristics, we deleted three items with a lower load and left four items for further analysis. According to the sample characteristics, we found that the three items were deleted as they were solely focused on service-based jobs and were not relevant to the larger sample. After deletion, the remaining items still have good reliability (*α* = 0.923). The remaining four items are consistent with the scale used by Farmer et al. (2003), which is proved to be more consistent with the measurement of Chinese employee sample.

#### Workplace Status

As a concept that reflects respect and status in organizations, most of the previous studies used employee subjective evaluation to measure workplace status. However, Blader and Yu (2017) argued that workplace status also has social consensus, which is often neglected in the measurement, and this often leads to the inaccuracy of the research. To avoid this potential inaccuracy, we invited the supervisors to assess their subordinates’ workplace status from the perspective of the observer, using the five items developed by Djurdjevic et al. (2017). A sample item is, “He or she has a great deal of prestige in the organization” (*α* = 0.909).

#### Control Variables

In line with previous studies (Marr et al., 2019; Bai et al., 2020), we mainly controlled the demographic variables of gender (1 = male, 2 = female), age (1 = age under 25, 2 = 26–30 years old, 3 = 31–40 years old, 4 = 41–50 years old, and 5 = age over 50), education (1 = high school and below, 2 = Junior college, 3 = Bachelor, 4 = Master, and 5 = Doctor), and work tenure (1 = within 1 year, 2 = 1–3 years, 3 = 4–6 years, 4 = 7–10 years, and 5 = more than 11 years).

### Data Analysis

In this study, we used SPSS22 to conduct correlation analysis, average calculation, SD calculation, and reliability analysis. To verify the distinctive validity among main research variables, we also conducted a CFA. On the one hand, we deleted unreasonable questionnaire items through CFA. On the other hand, by comparing the research model with the competition model, the discriminative validity of the variables in the model was tested. For the examination of mediation, moderation, and moderated mediation, we adopted Mplus7 to verify the moderated mediating model with path analysis. According to Preacher and Hayes (2008), we examined all the hypotheses simultaneously, to draw a more accurate research conclusion. To verify Hypothesis 2 and Hypothesis 4, we examined the indirect effects of strengths-based job crafting on employee creativity through job self-efficacy, with the bootstrap method using Mplus7.

## Results

### Confirmatory Factor Analysis

We conducted CFAwith Lisrel 8.8 to verify the distinctiveness among the four main variables in this study. Based on the results of the Chi-square test in Table 1, the measurement model of four factors exhibited a better fit with the data (*χ*^2^ = 289.77, df = 129, *χ*^2^/df = 2.246, CFI = 0.98, NFI = 0.97, IFI = 0.98, GFI = 0.93, RMSEA = 0.055, and SRMR = 0.051) than the other models. Within the measurement model, the standardized factor loadings ranged between 0.68 and 0.94, suggesting a good validity for the measurement.

**Table 1:** Results of the confirmatory factor analysis (CFA).

*Variable*	*χ^2^*	*df*	*χ^2^/df*	*SRMR*	*RMSEA*	*NFI*	*CFI*	*IFI*	*GFI*
Four-factor model (SJC, JSE,WP, and EC)	289.77	129	2.246	0.051	0.055	0.97	0.98	0.98	0.93
Three-factor model (SJC, JSE + WP, and EC)	1420.34	132	10.760	0.130	0.153	0.88	0.89	0.89	0.73
Two-factor model (SJC + JSE + WP, EC)	2912.32	134	21.734	0.190	0.223	0.79	0.80	0.80	0.56
Single-factor model (SJC, JSE,WP, and EC)	3864.68	135	28.627	0.180	0.257	0.71	0.72	0.72	0.49
Four-factor model + Method	246.37	92	2.678	0.028	0.064	0.97	0.98	0.98	0.93

*N* = 418.

SJC, strengths-based job crafting; JSE, job self-efficacy; WP, workplace status; EC, employee creativity; NFI, normed fit index; CFI, comparative fit index; IFI, incremental fit index; GFI, goodness of fit index; SRMR, standardized root mean square residual; and RMSEA, root mean square error of approximation.

### Descriptive Statistics and Tests of the Measurement Model

Table 2 presents means, SDs, correlations, convergent validity estimates (AVEs), and discriminant validity estimates (the square root of the AVEs) for the variables. The results show that all the correlation coefficients related to the research supposition are not larger than 0.6, suggesting a good discrimination validity among the main variables in the current study. As shown in Table 1, job crafting is positively related to job-based self-efficacy (*r* = 0.401, *p* < 0.01), employee creativity (*r* = 0.439, *p* < 0.01), and workplace status (*r* = 0.127, *p* < 0.01). There is a significant relationship between job self-efficacy and employee creativity (*r* = 0.489, *p* < 0.01).

**Table 2:** Means, SD, correlations, average variances extracted values, and tests of discriminant validity for the variables.

*Variables*	*Mean*	*SD*	*AVE*	*1*	*2*	*3*	*4*	*5*	*6*	*7*	*8*
(1) Gender	1.533	0.546	—								
(2) Age	2.394	0.861	—	−0.020							
(3) Education	3.063	0.574	—	0.098^*^	0.008						
(4) Work tenure	2.700	1.167	—	0.052	0.588^**^	0.017					
(5) JC-strengths	5.566	0.992	0.758	−0.053	0.173^**^	−0.054	0.077	**0.871**			
(6) Job self-efficacy	4.962	1.268	0.689	−0.024	0.027	0.021	−0.062	0.401^**^	**0.830**		
(7) Employee creativity	5.018	1.164	0.680	−0.020	0.069	−0.037	0.008	0.439^**^	0.489^**^	**0.825**	
(8) Workplace status	4.998	1.091	0.680	0.012	0.051	0.058	0.028	0.127^**^	0.240^**^	0.238^**^	**0.825**

*N* = 418. Bold figures on the diagonals are the square root of the average variances extracted values.

* *p* < 0.05;

** *p* < 0.01; ***p < 0.001.

Furthermore, we use the AVEs to assess the convergent validity of our four variables. All estimates were above the recommended value of 0.50 (Fornell and Larcker, 1981), and the square root of the AVE for each variable was significantly greater than its correlations with the other variables (Table 2). This further confirmed that there is a good discriminative validity among the main variables in this study.

### Common Method Variance

In the research process, supervisors’ evaluations of subordinates and subordinates’ self-evaluations were used to reduce the common method bias. Harman’s single-factor test generated four factors and the maximum variance contribution of the common factor was 22.869%, much less than half of the cumulative interpretation variance of 76.025%. In order to further eliminate common method deviation, this study uses the single method-factor approaches to test whether there is common method deviation (Xiong et al., 2012), as shown in Table 1. The results show that after adding the method factor, the fitting index of RMSEA, NFI, IFI, and GFI models is not significantly improved. Therefore, there is no serious common method bias in this study.

### Hypothesis Testing

In order to test the hypotheses, we followed the procedures proposed by Preacher and Hayes (2008) to test the indirect influence of strengths-based job crafting on employee creativity *via* job self-efficacy. As shown in Table 3, after control over the effects of gender, age, education, and work tenure, strengths-based job crafting has a significant impact on employee creativity (*B* = 0.333, *p* < 0.001). That is, the more behaviors of strengths-based job crafting, the more likely to inspire employee creativity. Hypothesis 1 is supported. Similarly, there is a positive correlation between strengths-based job crafting and job self-efficacy (*B* = 0.490, *p* < 0.001). As job self-efficacy is significantly related to employee creativity (*B* = 0.345, *p* < 0.001), the mediating effect of job self-efficacy has been confirmed. Therefore, Hypothesis 2 is supported.

**Table 3:** Mediation and moderation effects.

*Variables*	*Employee creativity*				*Job self-efficacy*			
	*B*	*SE*	*95%CI*		*B*	*SE*	*95%CI*	
			*LLCI*	*ULCI*			*LLCI*	*ULCI*
Intercept	3.420^***^	0.376	2.681	4.159	5.075^***^	0.365	4.357	5.792
Gender	0.012	0.089	−0.163	0.186	0.001	0.103	−0.201	0.203
Age	0.008	0.070	−0.13	0.146	0.014	0.081	−0.145	0.173
Education	−0.058	0.085	−0.224	0.108	0.048	0.098	−0.144	0.240
Work tenure	0.008	0.052	−0.093	0.110	−0.120*	0.059	−0.237	−0.004
Strengths-based job crafting	0.333^***^	0.054	0.228	0.439	0.490^***^	0.057	0.377	0.603
Job self-efficacy	0.345^***^	0.042	0.263	0.427				
Workplace status					0.239^***^	0.052	0.137	0.340
Strengths-based job crafting × Workplace status					0.140^**^	0.046	0.050	0.230

*N* = 418.

*p < 0.05;

** *p* < 0.01;

*** *p* < 0.001.

For the moderating effect of workplace status in the relationship between strengths-based job crafting and job self-efficacy, we adopted the procedures for testing a moderating effect developed by Hayes (2015). The analysis results of the moderating effect are presented in Table 3. As shown in Table 3, the interaction term of strengths-based job crafting and workplace status is significant in predicting employee creativity (*B* = 0.140, *p* < 0.01).

Considering the nesting between employees and supervisors in our study, we conduct the regression analysis clustering at the supervisor level to correct the SEs of individual-level analysis, as displayed in Table 4. As shown by Models 2, 3, and 6, job self-efficacy could partly mediate the influence of strengths-based job crafting on employee creativity. After aggregation to the team level, the moderating effect of workplace status on the relationship between strengths-based job crafting and job self-efficacy decreased to 0.108, but still significant (Model 7: *B* = 108, *p* < 0.001). Hence, Hypothesis 2 and Hypothesis 3 are well supported.

**Table 4:** Hierarchical regressions for main study variables.

Variables	Employee creativity				Job self-efficacy		
	M1	M2	M3	M4	M5	M6	M7
Gender	0.011	0.042	0.037	0.035	−0.043	−0.002	−0.002
Age	0.130	0.051	0.045	0.037	0.068	0.000	−0.015
Education	−0.048	−0.029	−0.056	−0.062	0.055	0.075	0.046
Work tenure	−0.044	−0.034	−0.009	−0.009	−0.065	−0.059	−0.066
Strengths-based job crafting		0.391^***^	0.256^***^	0.248^***^		0.383^***^	0.363^***^
Job self-efficacy			0.364^***^	0.343^***^			
Workplace status				0.115^**^			0.224^***^
Strengths-based job crafting × Workplace status				0.001			0.108^**^
*σ* ^2^	0.751	0.693	0.622	0.618	0.728	0.652	0.619
*τ*_00_	0.259	0.137	0.089	0.086	0.272	0.184	0.172

*p < 0.05;

** *p* < 0.01;

*** *p* < 0.001.

**Figure 2:**
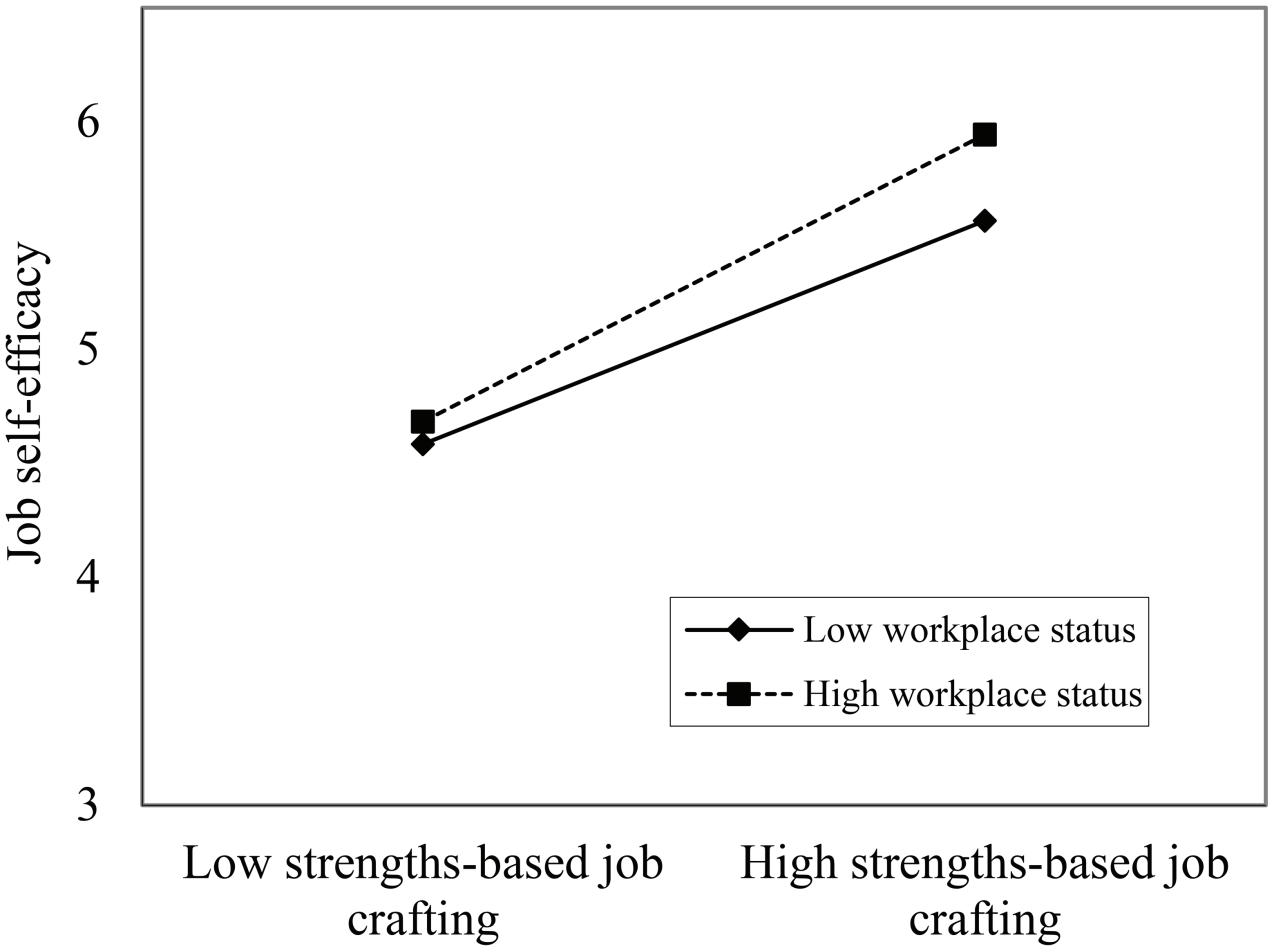
Moderating effect of workplace status in job crafting toward strengths and job self-efficacy.

Furthermore, to express the moderating effect of workplace status more vividly, a pitch diagram of the relationship between strengths-based job crafting and job self-efficacy had been displayed in Figure 2. The results confirmed that the influence of strengths-based job crafting on job self-efficacy is significantly moderated by the workplace status of the employees. The positive relationship between strengths-based job crafting and job-self efficacy is significantly stronger when workplace status is high than when it is low. Therefore, Hypothesis 3 received further support.

Hypothesis 4 proposed that workplace status moderated the indirect relationship between strengths-based job crafting and employee creativity *via* job-based self-efficacy. We used Mplus7 to conduct a bootstrap analysis to obtain the conditional indirect effect under the influence of workplace status (see Table 5). According to the results, the index of the moderating effect of workplace status on the indirect relationship between is 0.159, and the CI is [0.101, 0.234] does not include zero. Therefore, workplace status has a moderating effect on the indirect effect of strengths-based job crafting and employee creativity. Thus, Hypothesis 4 is supported.

**Table 5:** Moderated mediation effect.

*Moderate variance*	*Strengths-based job crafting → Job self-efficacy → Employee creativity*								
	*Direct effect*				*Indirect effect*			
	*Effect*	*SE*	*LLCI*	*ULCI*	*Effect*	*SE*	*LLCI*	*ULCI*
High workplace status (M + 1SD)	0.289	0.072	0.148	0.430	0.209	0.043	0.135	0.304
Middle workplace status (M)	0.325	0.053	0.220	0.430	0.159	0.034	0.101	0.234
Low workplace status (M-1SD)	0.361	0.066	0.231	0.491	0.109	0.036	0.049	0.191

## Discussion

Strength is a hot topic in the field of employee self-management (Miglianico et al., 2020; Bhatnagar et al., 2021), and its influence on employee performance has attracted the attention of scholars and managers. Based on the literature review, we examined why and when strengths-based job crafting can promote employee creativity. Through the mediating effect of job self-efficacy and the moderating effect of workplace status, we explored the relationship between strengths-based job crafting and employee creativity. In this study, a total number of 87 questionnaires for supervisors and 418 questionnaires for subordinates were used to test our hypothesis. Results indicated that strengths-based job crafting can promote employee creativity through job self-efficacy, and these findings were more salient when the employees perceived a higher workplace status.

### Theoretical Implications

Our findings in this study contribute to the literature on strengths-based job crafting and employee creativity in several ways. First, this study confirms that strengths-based job crafting is an important antecedent variable of employee creativity. The job crafting literature has provided several theoretical perspectives to explain how job crafting influences individual creativity, such as job demands and resource allocation (Beck and Schmidt, 2018; Sun et al., 2020). However, these studies have mainly focused on the “job” part, and the “person” part have been largely overlooked. Distinct from top-down job crafting, strengths-based job crafting is a new type of bottom-up job crafting style to make better use of personal strengths. Although scholars have pointed to the importance of strength use in the workplace, studies toward exploring the influence of personal strengths on performance only recently begun to attract attention (Miglianico et al., 2020). Furthermore, these studies are primarily limited to focusing on the characteristics of personal strengths, and do not explore the psychological mechanism of influence. Therefore, we move beyond previous research by revealing the psychological self-affirmation process between strengths-based job crafting and employee creativity, extending the application of self-affirmation theory to the work context, and enriching the existing literature on self-affirmation theory.

Second, we revealed an influencing mechanism that transmits the effect of strengths-based job crafting on employee creativity. As indicated by many scholars, a simultaneous effect of self-and social systems determined the formation process of employee creativity (Dierdorff and Jensen, 2018; Sun et al., 2020). Therefore, our study introduced job self-efficacy as a mediator variable in the theoretical model. The results suggested that job self-efficacy significantly mediates the relationship between strengths-based job crafting and employee creativity. Our findings would not only expand the theoretical research on the relationship between strength and creativity but also enrich the research on individual internal belief systems related to creativity. To some extent, our work also responds to the call of Kooij et al. (2017) and opens the “black box” in the process of strengths-based job crafting influencing employee creativity.

Furthermore, not only do we examine a potential consequence of strengths-based job crafting on employee creativity, but we also examine when and why this consequence occurs. From a social enhancement perspective, we found that the self-affirmation process caused by strengths-based job crafting will be affected by the coworkers’ subjective assessment. The results suggest that workplace status can positively moderate the relationship between strengths-based job crafting and employee creativity. For employees with a higher workplace status, strengths-based job crafting may generate more beliefs to promote employee creativity. More specifically, the finding that the conditional indirect influence of strengths-based job crafting on employee creativity through job self-efficacy, differs in different workplace status.

### Practical Implications

Our results suggest that employees who engaged in strengths-based job crafting are more likely to present new ideas. By exploring the influence of strengths-based job crafting on employee creativity, this study provides s practical reference value for organizations to leverage employees’ personal strengths better. Enterprise managers can establish strength discovery and incentive mechanisms to stimulate the formation of creativity. In addition, the enterprise should provide employees with appropriate work characteristics, which can help to enhance the exertion of personal strength in the workplace. This study also found that job self-efficacy plays a mediating role, which requires the organization must pay attention to the employees’ personal values and beliefs (Cohen and Sherman, 2014; Mao et al., 2021). When individuals look at themselves from the perspective of strength and redesign their work to make a better use of the personal strengths, they will develop a growth mode of thinking and become more confident to put forward new ideas. Therefore, managers should be attentive to preserving employees’ job self-efficacy by fostering positive stimuli related to their strengths. When job self-efficacy was preserved, employees’ self-and social systems will lead to the enhancement of self-affirmation and the generation of creativity. In addition, this study further shows that the extent to which strengths-based job crafting potentially aid employee creativity performance is largely dependent on the work context. Workplace status plays an important role in fostering positive stimulate and facilitating favorable creative performance. Employees are more likely to present new and useful ideas if they perceived a higher workplace status. Therefore, focusing on implementing clear and consistent performance metrics and creating a culture where team members’ workplace status can be respected and valued becomes especially important.

### Limitations and Future Research

Despite the implications above, our research has several potential limitations inevitably, some of which may inspire future research. From the perspective of research design, although the data were collected from two sources at two different times to control the common method bias (Podsakoff et al., 2003); the measurements of strengths-based job crafting, job self-efficacy, workplace status, and employee creativity were still measured by using participants’ subjective perception. In addition, the impact of workplace status on employees is a long-term dynamic process (Anderson et al., 2015; Djurdjevic et al., 2017), so questionnaires cannot strictly measure the relationship between related variables. Therefore, this study encourages future researchers to adopt an experimental design to draw clear conclusions about causality.

Second, this study constructed and verified a model to examine the internal mechanism of the relationship between strengths-based job crafting and employee creativity, as well as the boundary condition of the relationship. However, this study only introduced job self-efficacy as a mediator in the relationship, ignoring the other replaceable variables which can explain the management phenomenon. Therefore, future studies can explore this topic from different theoretical perspectives to deepen the understanding of this management problem.

Finally, this study only discusses the moderating effect of workplace status on the mechanism of job self-efficacy. We encourage future studies to investigate employee strength within a team context. Exploring how personal strength influence the interaction between individuals with their team members can yield interesting results. For example, to the extent that employees have regular interactions with leaders, colleagues, and customers, the perceptions of these groups may be important for understanding both the development, as well as outcomes, of their strengths.

## Data Availability Statement

The raw data supporting the conclusions of this article will be made available by the authors, without undue reservation.

## Author Contributions

ZY designed the study, collected the data, performed the data analysis, drafted the manuscript, proofread the manuscript, and validated the results. PL and KC drafted, reviewed, and revised the manuscript. All authors contributed to the article and approved the submitted version.

## Funding

This research is supported and funded by the National Social Science Fund of China (Grant No. 20BGL163).

## Conflict of Interest

The authors declare that the research was conducted in the absence of any commercial or financial relationships that could be construed as a potential conflict of interest.

## Publisher’s Note

All claims expressed in this article are solely those of the authors and do not necessarily represent those of their affiliated organizations, or those of the publisher, the editors and the reviewers. Any product that may be evaluated in this article, or claim that may be made by its manufacturer, is not guaranteed or endorsed by the publisher.
